# Supplementary material for: Learning deep abdominal CT registration through adaptive loss weighting and synthetic data generation
Source: PLoS One. 2023 Feb 24;18(2):e0282110. doi: 10.1371/journal.pone.0282110 (PMC9956065; doi:10.1371/journal.pone.0282110)
Supplement: S1 Appendix — Additional details about the segmentations produced for the IXI dataset. (PDF) [file pone.0282110.s001.pdf]

S1: Additional data details

Javier Pérez de Frutos[1*], André Pedersen[1,2,3], Egidijus Pelanis[4], David Bouget[1], Shanmugapriya Survarachakan[5], Thomas Langø[1,6], Ole-Jakob Elle[4], and Frank Lindseth[5]

[1]Department of Health Research, SINTEF, Trondheim, Norway
[2]Department of Clinical and Molecular Medicine, Norwegian University of Science and University (NTNU), Trondheim, Norway
[3]Clinic of Surgery, St. Olavs hospital, Trondheim University Hospital, Trondheim, Norway
[4]Intervention Centre, Oslo University Hospital, Oslo, Norway
[5]Department of Computer Science, Norwegian University of Science and University (NTNU), Trondheim, Norway
[6]Research Department, Future Operating Room, St. Olavs hopsital, Trondheim University Hospital, Trondheim, Norway
[*]Corresponding author: Javier Pérez de Frutos, javier.perezdefrutos@sintef.no

Document description

This document contains additional details about the segmentations produced for the IXI dataset. A total of 28 regions where segmented on the IXI dataset using the MNI 152 space atlas. From these, 24 classes distinguished between left and right sections, which were merged together resulting in a collection of 16 labels. A total of 17 labels, including the background, were used. For more details, see Table A.

Table A. Labels used from the MNI 152 space atlas.

Parietal grey matter (left and right)	Fornix (left and right)
Lateral ventricle (left and right)	Caudate (left and right)
Occipital grey matter (left and right)	Cerebellum (left and right)
Globus pallidus (left and right)	Thalamus (left and right)
Putamen (left and right)	Frontal grey matter (left and right)
Subthalamic nucleus (left and right)	Temporal grey matter (left and right)
3rd ventricle	Brain stem
4th ventricle	Cerebrospinal fluid
	Background
