# Supplementary material for: Learning deep abdominal CT registration through adaptive loss weighting and synthetic data generation
Source: PLoS One. 2023 Feb 24;18(2):e0282110. doi: 10.1371/journal.pone.0282110 (PMC9956065; doi:10.1371/journal.pone.0282110)
Supplement: S2 Appendix — Details on the GPU resources usage by the proposed augmentation layer. (PDF) [file pone.0282110.s002.pdf]

S2: Resources impact of the augmentation layer

Javier Pérez de Frutos[1*], André Pedersen[1,2,3], Egidijus Pelanis[4], David Bouget[1], Shanmugapriya Survarachakan[5], Thomas Langø[1,6], Ole-Jakob Elle[4], and Frank Lindseth[5]

[1]Department of Health Research, SINTEF, Trondheim, Norway
[2]Department of Clinical and Molecular Medicine, Norwegian University of Science and University (NTNU), Trondheim, Norway
[3]Clinic of Surgery, St. Olavs hospital, Trondheim University Hospital, Trondheim, Norway
[4]Intervention Centre, Oslo University Hospital, Oslo, Norway
[5]Department of Computer Science, Norwegian University of Science and University (NTNU), Trondheim, Norway
[6]Research Department, Future Operating Room, St. Olavs hopsital, Trondheim University Hospital, Trondheim, Norway
[*]Corresponding author: Javier Pérez de Frutos, javier.perezdefrutos@sintef.no

Document description

This document contains details on the GPU resources usage by the proposed augmentation layer. Figs. Aa and Ab which show the GPU usage during one training epoch. The relative increase in time is of 7.7% (ca. 10.5 s). Fig. Ac shows the memory usage on the GPU during one training epoch with and without using the augmentation layer. The increase in memory allocation is of 0.47% (ca. 74 MB / 16 GB).

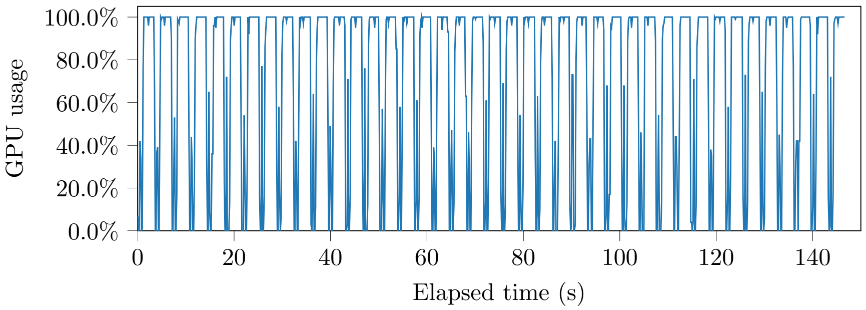

(a) GPU usage with augmentation layer

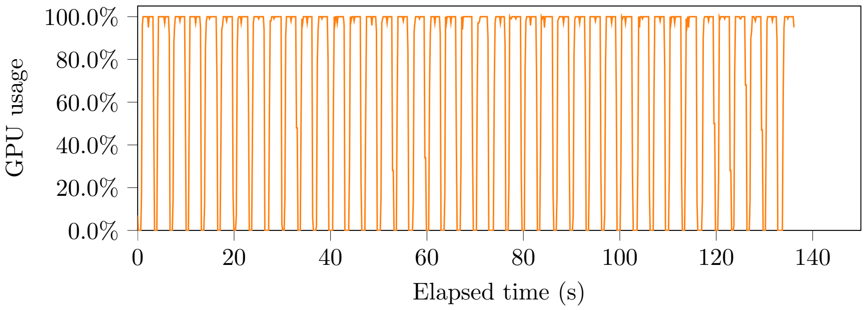

(b) GPU usage without augmentation layer

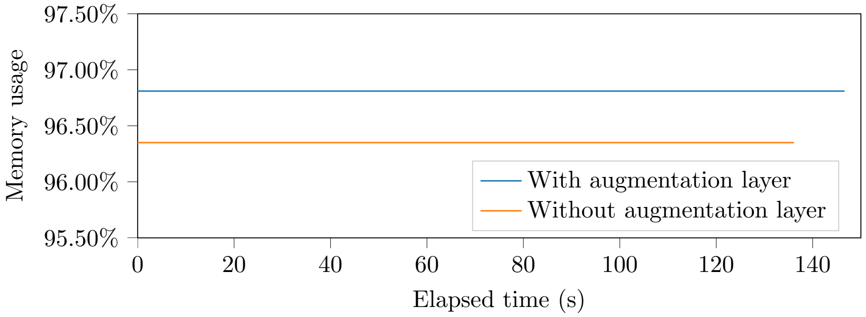

(c) Memory usage with (blue) and without using the augmentation layer (orange)

Figure A. Impact of the augmentation layer on the GPU resources during one training epoch.
