# Supplementary material for: Learning deep abdominal CT registration through adaptive loss weighting and synthetic data generation
Source: PLoS One. 2023 Feb 24;18(2):e0282110. doi: 10.1371/journal.pone.0282110 (PMC9956065; doi:10.1371/journal.pone.0282110)
Supplement: S3 Appendix — Figures of the training curves, as well as the adaptive loss weighting. (PDF) [file pone.0282110.s003.pdf]

S3: Training curves

Javier Pérez de Frutos[1*], André Pedersen[1,2,3], Egidijus Pelanis[4], David Bouget[1], Shanmugapriya Survarachakan[5], Thomas Langø[1,6], Ole-Jakob Elle[4], and Frank Lindseth[5]

[1]Department of Health Research, SINTEF, Trondheim, Norway
[2]Department of Clinical and Molecular Medicine, Norwegian University of Science and University (NTNU), Trondheim, Norway
[3]Clinic of Surgery, St. Olavs hospital, Trondheim University Hospital, Trondheim, Norway
[4]Intervention Centre, Oslo University Hospital, Oslo, Norway
[5]Department of Computer Science, Norwegian University of Science and University (NTNU), Trondheim, Norway
[6]Research Department, Future Operating Room, St. Olavs hopsital, Trondheim University Hospital, Trondheim, Norway
[*]Corresponding author: Javier Pérez de Frutos, javier.perezdefrutos@sintef.no

Document description

This document contains figures showing the training curves, as well as figures related to the adaptive loss weighting.

Training curves

Figs. A - D show training and validation losses for the six different configurations described in the main manuscript, for the IXI and Oslo-CoMet datasets (see Table 1, Section 2.5). Furthermore, training runtime of 160 epochs is shown as part of the legends of both figures for reference.

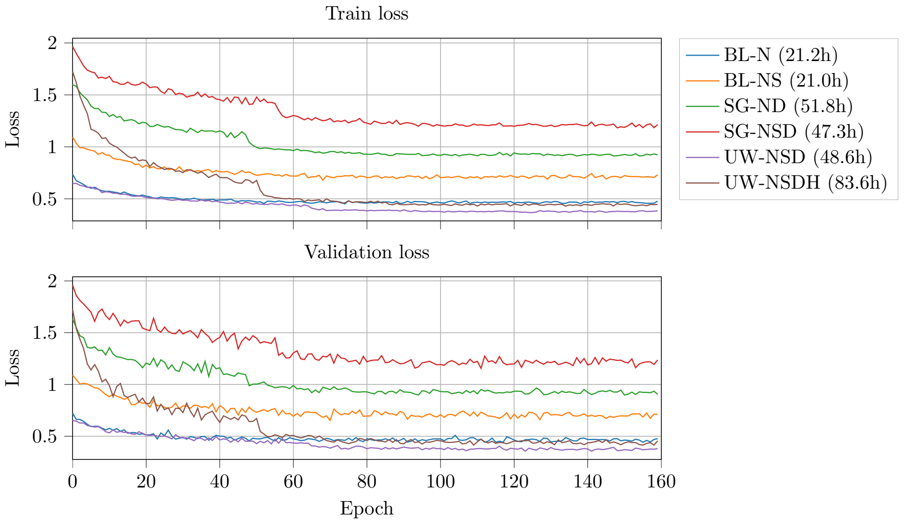

Figure A. Train and validation loss of the models trained on the IXI dataset.

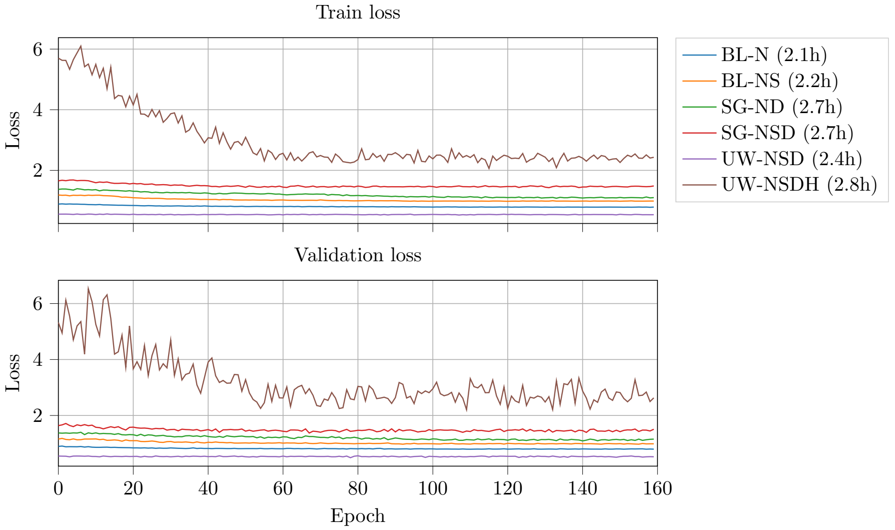

Figure B. Train and validation loss of the models trained on the Oslo-CoMet dataset.

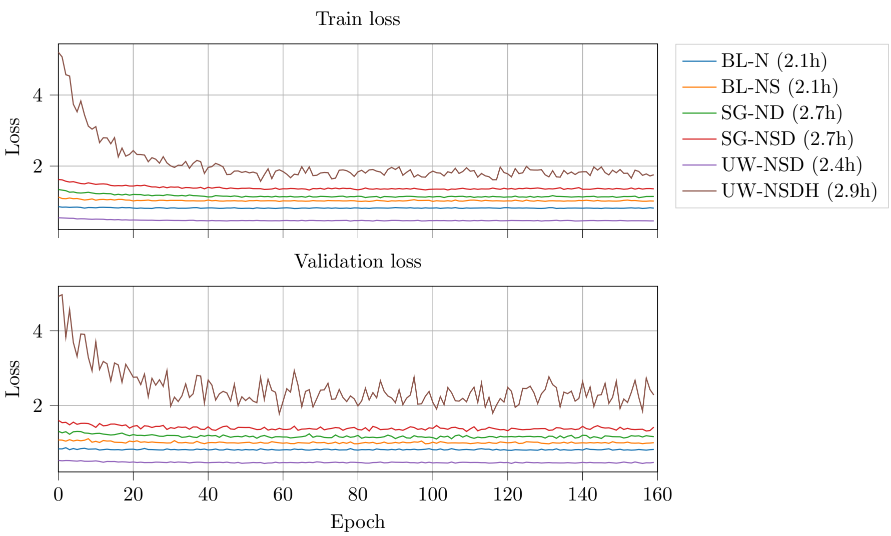

Figure C. Train and validation loss of the models trained on the Oslo-CoMet dataset from finetuning the entire architecture.

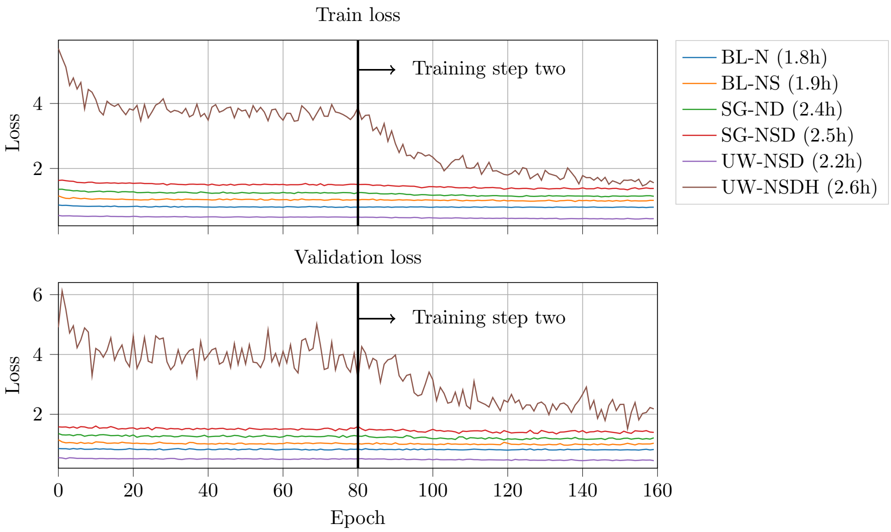

Figure D. Train and validation loss of the models trained on the Oslo-CoMet dataset from finetuning in two steps.

Adaptive loss weighting results

Figs. E - L show the evolution of the dynamic weight in the UW models for the studied cases.

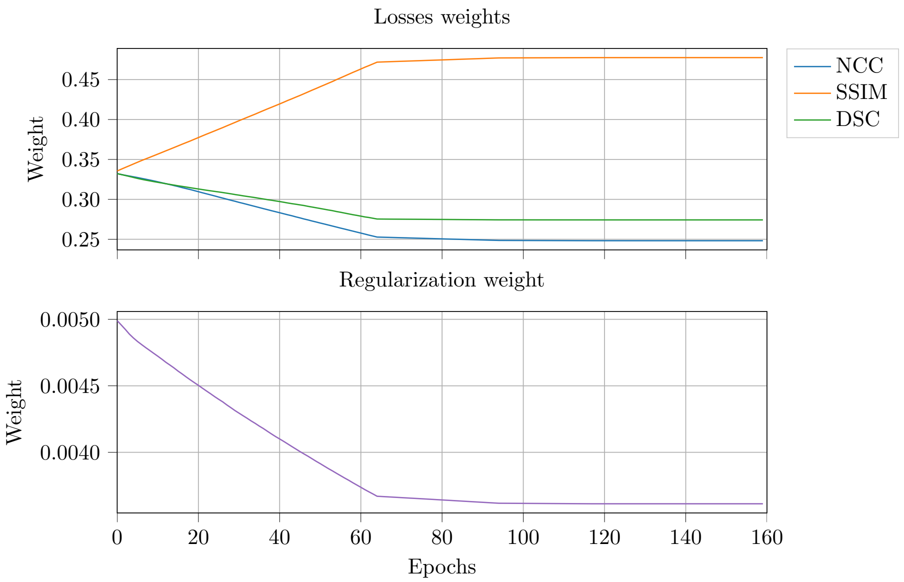

Figure E. Loss weights of the model UW-NSD trained on the IXI dataset.

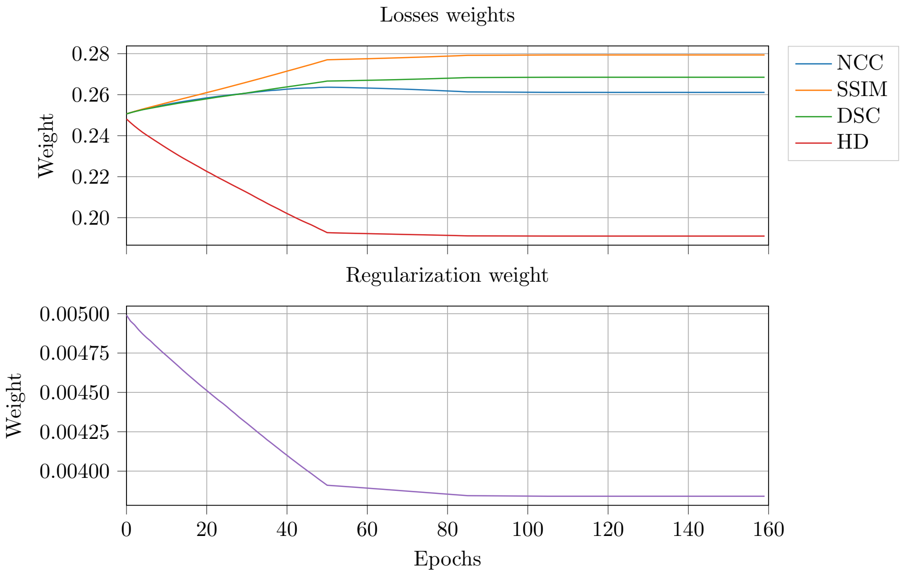

Figure F. Loss weights of the model UW-NSDH trained on the IXI dataset.

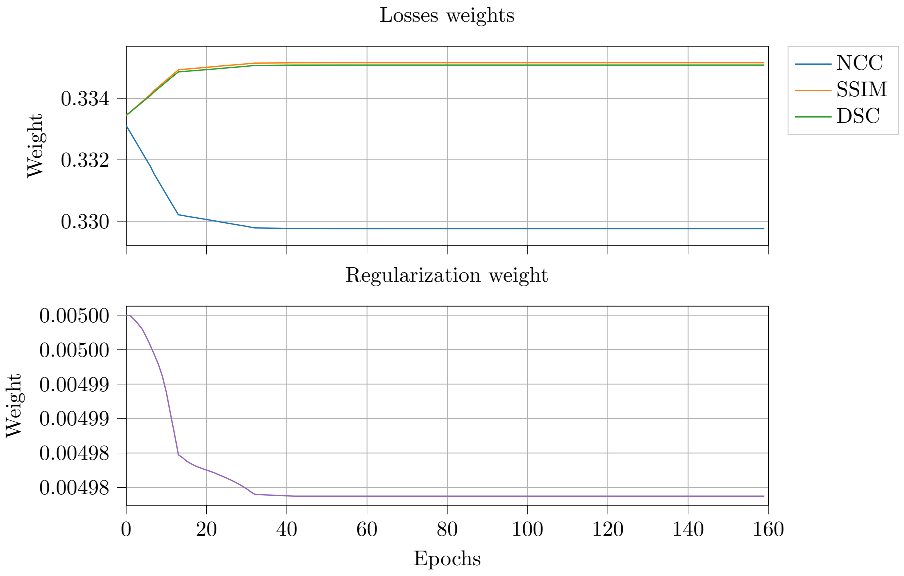

Figure G. Loss weights of the model UW-NSD trained on the Oslo-CoMet dataset.

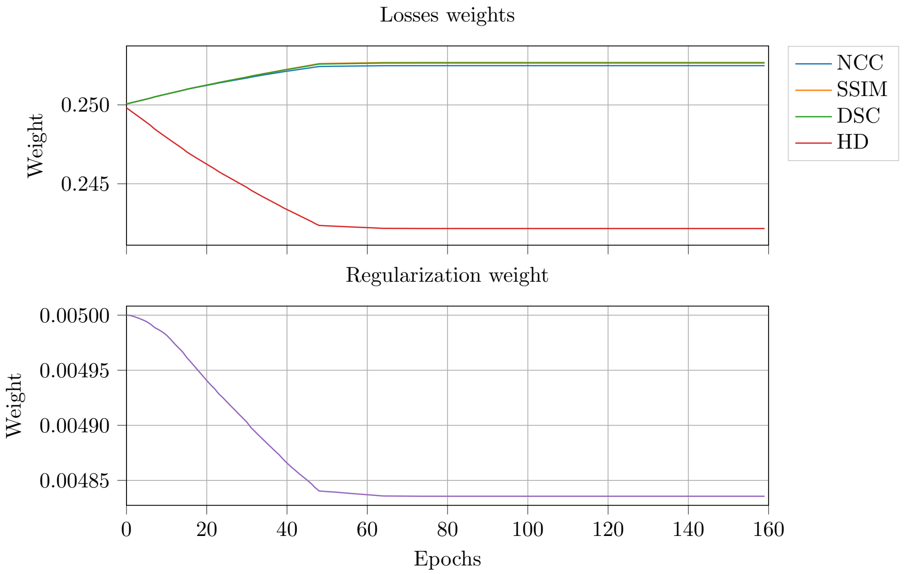

Figure H. Loss weights of the model UW-NSDH trained on the Oslo-CoMet dataset.

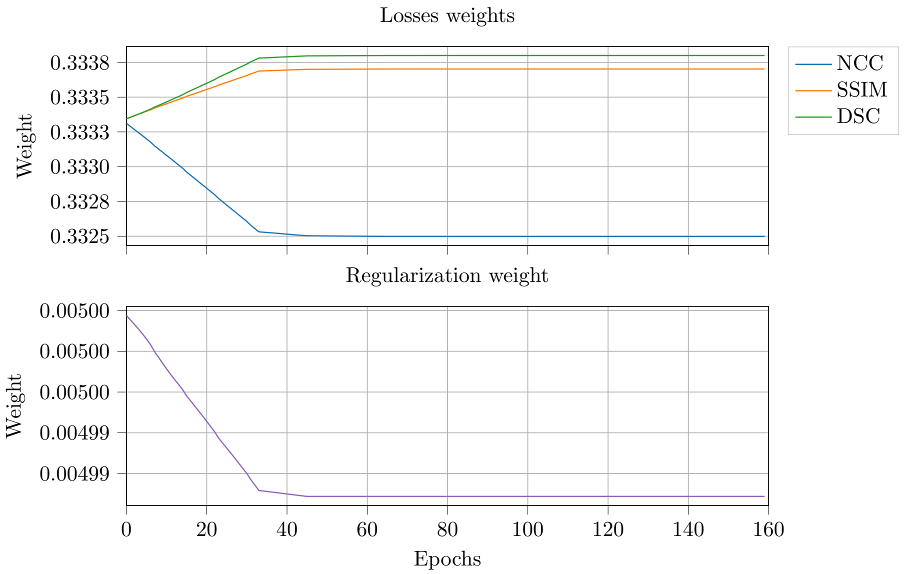

Figure I. Loss weights of the model UW-NSD trained on the Oslo-CoMet dataset from finetuning the entire architecture.

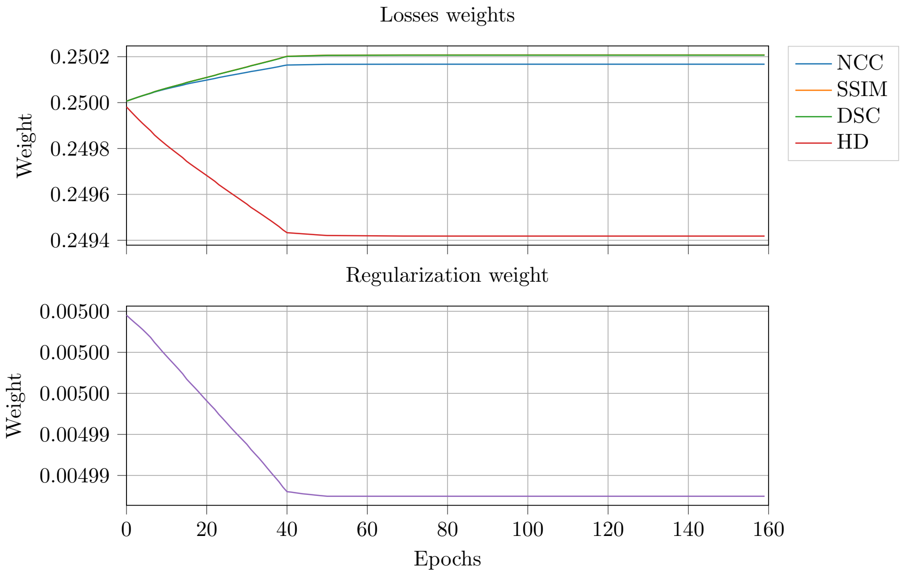

Figure J. Loss weights of the model UW-NSDH trained on the Oslo-CoMet dataset from finetuning the entire architecture.

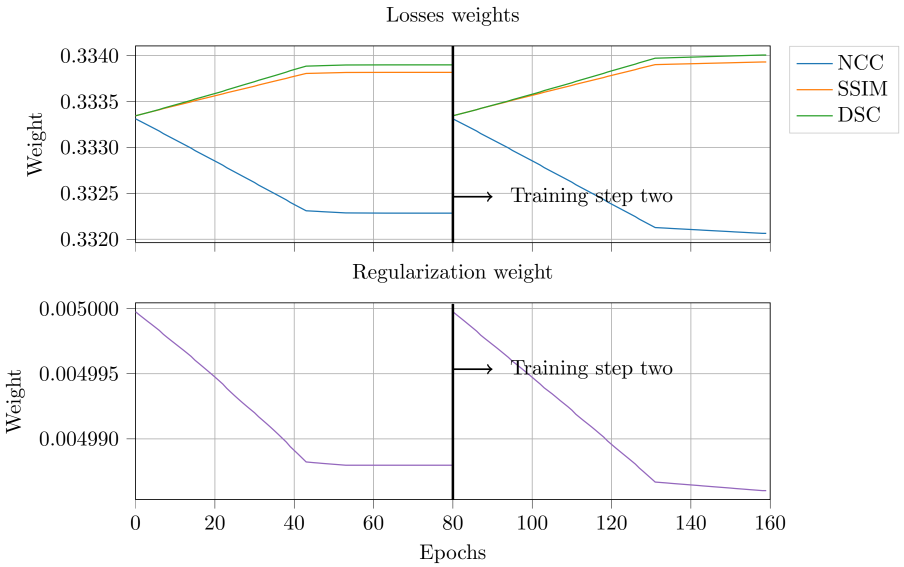

Figure K. Loss weights of the model UW-NSD trained on the Oslo-CoMet dataset from finetuning in two steps.

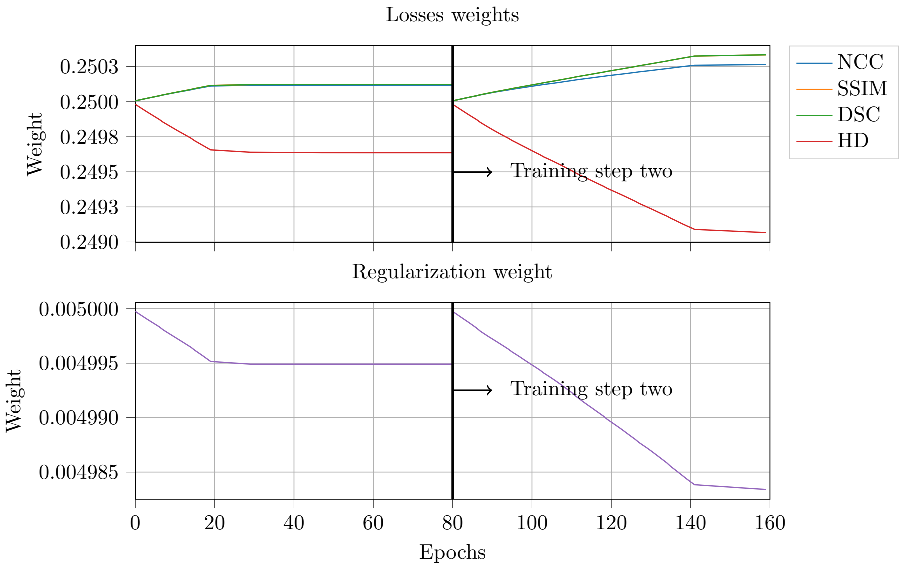

Figure L. Loss weights of the model UW-NSDH trained on the Oslo-CoMet dataset from finetuning in two steps.
