# Supplementary material for: Learning deep abdominal CT registration through adaptive loss weighting and synthetic data generation
Source: PLoS One. 2023 Feb 24;18(2):e0282110. doi: 10.1371/journal.pone.0282110 (PMC9956065; doi:10.1371/journal.pone.0282110)
Supplement: S4 Appendix — Results of the statistical tests described in the manuscript, and additional comparison between manually and automatically generated segmentations. (PDF) [file pone.0282110.s004.pdf]

S4: Statistical analysis

Javier Pérez de Frutos[1*], André Pedersen[1,2,3], Egidijus Pelanis[4], David Bouget[1], Shanmugapriya Survarachakan[5], Thomas Langø[1,6], Ole-Jakob Elle[4], and Frank Lindseth[5]

[1]Department of Health Research, SINTEF, Trondheim, Norway
[2]Department of Clinical and Molecular Medicine, Norwegian University of Science and University (NTNU), Trondheim, Norway
[3]Clinic of Surgery, St. Olavs hospital, Trondheim University Hospital, Trondheim, Norway
[4]Intervention Centre, Oslo University Hospital, Oslo, Norway
[5]Department of Computer Science, Norwegian University of Science and University (NTNU), Trondheim, Norway
[6]Research Department, Future Operating Room, St. Olavs hopsital, Trondheim University Hospital, Trondheim, Norway
[*]Corresponding author: Javier Pérez de Frutos, javier.perezdefrutos@sintef.no

Document description

This document contains tables showing detailed results of the statistical tests described in the manuscript, and a comparison between manually and automatically generated segmentations.

Statistical analysis

Five sets of statistical tests were conducted to assess: 1) performance contrasts between model designs (see Table A), 2) benefit of transfer learning (see Table B), 3) benefit of segmentation-guiding, and 4) benefit of uncertainty weighting (see Table C), and 5) performance contrasts between the baseline and segmentation-guided models, and the traditional methods in ANTs (SyN and SyNCC) (see Table D). For the tests, The TRE metric was used, as it is considered the gold standard for surgical practitioners. Tests were conducted on the evaluations of the IXI and Oslo-CoMet test datasets only. For the tests only involving the Oslo-CoMet dataset, the two-step transfer learning approach was used as reference. A significance level of 5% was used to determine statistical significance.

Table A. Results of multiple comparisons comparing TRE between all designs evaluated on the IXI dataset.

	BL-NS	SG-ND	SG-NSD	UW-NSD	UW-NSDH
**BL-N**	0.9	<0.001	<0.001	<0.001	<0.001
**BL-NS**	-	<0.001	<0.001	<0.001	<0.001
**SG-ND**	-	-	0.9	0.9	0.9
**SG-NSD**	-	-	-	0.9	0.9
**UW-NSD**	-	-	-	-	0.9

Table B. Results of hypotheses tests assessing the benefit of transfer learning on the Oslo-CoMet dataset in terms of TRE for the three designs: BL-N, SG-NSD, and UW-NSD.

Model	p-value
BL-N	0.8608
SG-NSD	0.0021
UW-NSD	0.0014

Table C. Results of hypotheses tests assessing the added value of segmentation-guiding and uncertainty weighting on the Oslo-CoMet dataset.

Model	p-value
Segmentation-guiding	<0.001
Uncertainty-weighting	0.0093

Table D. Results of hypotheses tests assessing the performance of the baseline and segmentation-guiding models compared to traditional method ANTs (SyN and SyNCC approaches), on the Oslo-CoMet dataset.

AI Model	ANTs approach	p-value
BL-N	SyN	0.5845
	SyNCC	0.5845
SG-NSD	SyN	<0.001
	SyNCC	<0.001

Comparison between manual and automatic annotations

Manual and automatic segmentations of the parenchyma and the vascular structures were performed on the Oslo-CoMet test set images. In Table E the DSC and HD95 are reported.

Table E. DSC and HD95 comparing manual and automatic annotations on the Oslo-CoMet test set images.

Label	DSC	HD95
**Parenchyma**	0.946±0.046	10.122±11.032
**Vessels**	0.355±0.090	24.872±5.161
