# Supplementary material for: Learning deep abdominal CT registration through adaptive loss weighting and synthetic data generation
Source: PLoS One. 2023 Feb 24;18(2):e0282110. doi: 10.1371/journal.pone.0282110 (PMC9956065; doi:10.1371/journal.pone.0282110)
Supplement: S5 Appendix — Examples of predictions on the IXI and Oslo-CoMet test datasets. (PDF) [file pone.0282110.s005.pdf]

S5: Qualitative results

Javier Pérez de Frutos[1*], André Pedersen[1,2,3], Egidijus Pelanis[4], David Bouget[1], Shanmugapriya Survarachakan[5], Thomas Langø[1,6], Ole-Jakob Elle[4], and Frank Lindseth[5]

[1]Department of Health Research, SINTEF, Trondheim, Norway
[2]Department of Clinical and Molecular Medicine, Norwegian University of Science and University (NTNU), Trondheim, Norway
[3]Clinic of Surgery, St. Olavs hospital, Trondheim University Hospital, Trondheim, Norway
[4]Intervention Centre, Oslo University Hospital, Oslo, Norway
[5]Department of Computer Science, Norwegian University of Science and University (NTNU), Trondheim, Norway
[6]Research Department, Future Operating Room, St. Olavs hopsital, Trondheim University Hospital, Trondheim, Norway
[*]Corresponding author: Javier Pérez de Frutos, javier.perezdefrutos@sintef.no

Document description

This document contains examples of predictions on the IXI and Oslo-CoMet test datasets.

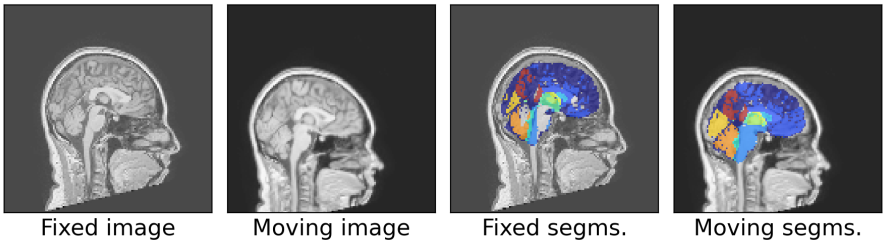

(a) Sample test image.

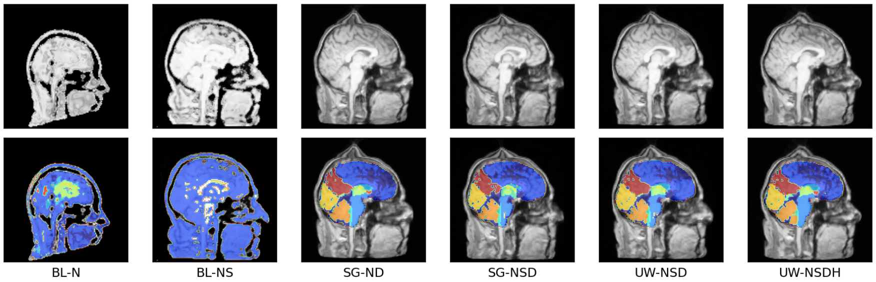

(b) Predictions of the evaluated models on the sample image.

Figure A. Sample image of the IXI test set, and predictions of the models.

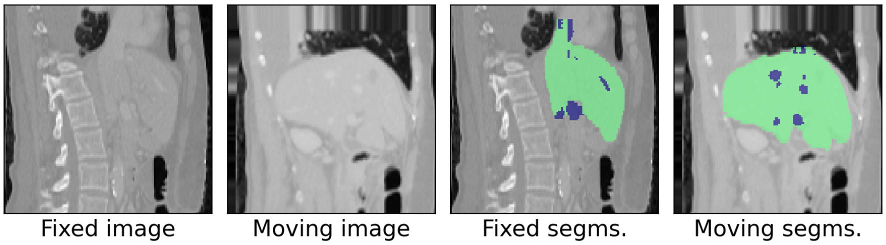

(a) Sample test image.

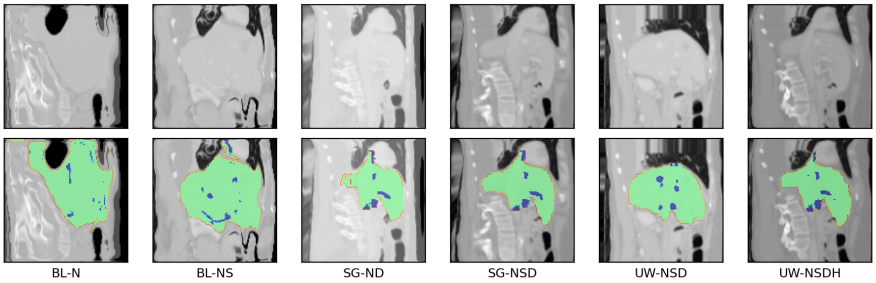

(b) Predictions of the models trained on the Oslo-CoMet dataset.

Figure B. Sample image of the Oslo-CoMet test set, and predictions of the models trained on the Oslo-CoMet dataset.

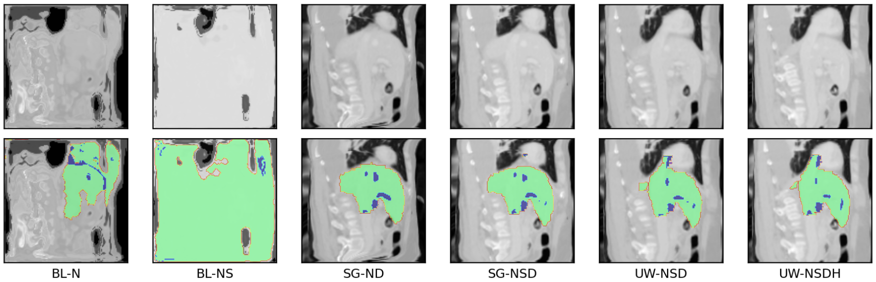

(a) Predictions of the models trained on the Oslo-CoMet dataset from finetuning the entire architecture.

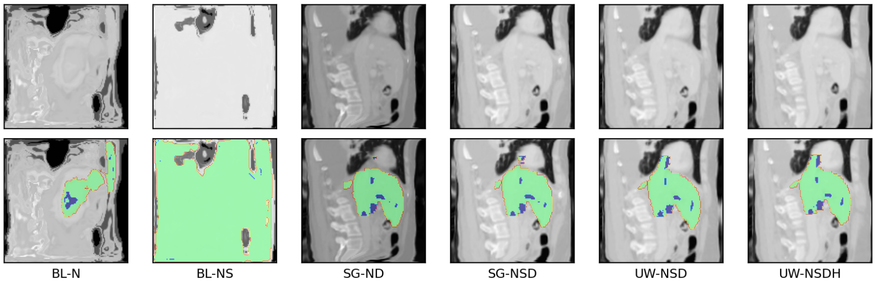

(b) Predictions of the models trained on the Oslo-CoMet dataset from finetuning in two steps.

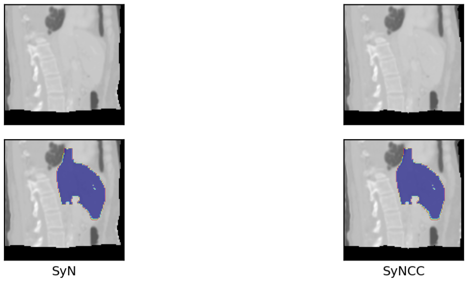

(c) Predictions of the SyN and SyNCC algorithms.

Figure C. Predictions of the finetuned models and ANTs on the Oslo-CoMet test set sample.
