# Supplementary material for: Learning deep abdominal CT registration through adaptive loss weighting and synthetic data generation
Source: PLoS One. 2023 Feb 24;18(2):e0282110. doi: 10.1371/journal.pone.0282110 (PMC9956065; doi:10.1371/journal.pone.0282110)
Supplement: Attachment — Submitted filename: Response to reviewers.pdf [file pone.0282110.s007.pdf]

Response letter for review on PONE-D-22-33384

Dear Paolo Cazzaniga, Academic Editor PLOS ONE,

We sincerely appreciate the work of the reviewers for our manuscript originally titled 'Train smarter, not harder: learning deep abdominal CT registration on scarce data', now titled 'Learning deep abdominal CT registration through adaptive loss weighting and synthetic data generation', by Pérez de Frutos, J., *et al.* with reference PONE-D-22-33384. The comments raised by the Editor and reviewers were valuable for us and have further improved the quality of the paper. We have made changes in the manuscript according to the suggestions. All changes have been addressed in detail in this letter.

We believe that all aspects of the paper have been improved, including, as required by the Editor, the discussion on the state-of-the-art, the description of the methodology, and the presentation of the experimental results. This rebuttal letter responds to each point raised by the academic editor and reviewer(s). Together with this letter, the following documents have been submitted:

- A marked-up copy of the manuscript and the Supplementary Materials with highlighted changes made to the original versions have been uploaded as separate files labelled 'Revised Manuscript with Track Changes' and 'Revised Supplementary Materials with Track Changes', respectively. Changes are shown in red.
- A clean version of our revised paper and Supplementary Materials without tracked changes have also been uploaded and labelled 'Manuscript' and 'Supplementary Materials', respectively.
- The file containing the numerical results obtained in this study has been included under the name 'evaluation_results.csv'.

In addition to the concerns raised by the Editor and the reviewers, we made the following changes to the original manuscript:

- The naming of the Oslo-CoMet dataset has been changed to match that of the original article [25]. This changes can be found in lines 81, 93, 99, 186, 196, 205, 225, 226, 232, 258, 263, 272, 339, 358; the titles of Tables 1, 3, 4, and 5; and throughout the provided Supplementary Materials.
- At the same time the new experiments were done, following the reviewers suggestions, we realised that the background segmentation was included in the evaluation pipeline. This resulted in bloated values for the DSC, Hausdorff distance, and target registration error. This defect was promptly corrected and so additional modifications were carried out, aside from those pertaining the addressed concerns. The corrected values have

been included in Tables 2 to 4. A clarification has been added to the manuscript (lines 212-213). S16 Figure, in the Supplementary Materials, has been updated. And both the Results and Discussion sections have been updated to reflect the corrected results (lines 272-277, and lines 335-344, respectively). Even though the metrics have changed, showing a better performance of the ANTs methods on the segmentation based metrics, the trends discussed in original submission were unaltered. We apologised for any inconvenience this might produce to the reviewers.

Journal requirements

Concern 1: Please ensure that your manuscript meets PLOS ONE's style requirements, including those for file naming. The PLOS ONE style templates can be found at `https://journals.plos.org/plosone/s/file?id=wjVg/PLOSOne_formatting_sample_main_body.pdf` and `https://journals.plos.org/plosone/s/file?id=ba62/PLOSOne_formatting_sample_title_authors_affiliations.pdf`.

Response: We have gone through the guideline provided, and rectified those elements which did not match the journal style, such as, titles of figures and tables, references to the online resources, the numbering on the affiliation of the authors, and the footnote of the corresponding author.

Concern 2: Please provide additional details regarding participant consent. In the ethics statement in the Methods and online submission information, please ensure that you have specified what type you obtained (for instance, written or verbal, and if verbal, how it was documented and witnessed). If your study included minors, state whether you obtained consent from parents or guardians. If the need for consent was waived by the ethics committee, please include this information. Once you have amended this/these statement(s) in the Methods section of the manuscript, please add the same text to the "Ethics Statement" field of the submission form (via "Edit Submission").

For additional information about PLOS ONE ethical requirements for human subjects research, please refer to `http://journals.plos.org/plosone/s/submission-guidelines#loc-human-subjects-research`.

Response: Modifications have been made to the Methods section (see lines 84-85, and 94-98). The ethical statements were added to the Ethics Statement field of the submission form.

Concern 3: We note that the grant information you provided in the 'Funding Information' and 'Financial Disclosure' sections do not match. When you re-

submit, please ensure that you provide the correct grant numbers for the awards you received for your study in the 'Funding Information' section.

Response: The funding from the second financial source had no grant number, hence we were unsure if to include it. This has been fixed now in the submission form.

Concern 4: Thank you for stating the following financial disclosure: "This study was supported by the H2020-MSCA-ITN Project No. 722068 HiPerNav; Norwegian National Advisory Unit for Ultrasound and Image-Guided Therapy (St. Olavs hospital, NTNU, SINTEF); SINTEF; St. Olavs hospital; and the Norwegian University of Science and Technology (NTNU)." Please state what role the funders took in the study. If the funders had no role, please state: "The funders had no role in study design, data collection and analysis, decision to publish, or preparation of the manuscript." If this statement is not correct you must amend it as needed. Please include this amended Role of Funder statement in your cover letter; we will change the online submission form on your behalf.

Response: The funders had no role in this study. Hence, *"The funders had no role in study design, data collection and analysis, decision to publish, or preparation of the manuscript"* has been added to the Cover Letter.

Concern 5: Please include your full ethics statement in the 'Methods' section of your manuscript file. In your statement, please include the full name of the IRB or ethics committee who approved or waived your study, as well as whether or not you obtained informed written or verbal consent. If consent was waived for your study, please include this information in your statement as well.

Response: We have made the necessary modifications to the Methods section, see lines 84-85 and 94-98.

Concern 6: Please include captions for your Supporting Information files at the end of your manuscript, and update any in-text citations to match accordingly. Please see our Supporting Information guidelines for more information: `http://journals.plos.org/plosone/s/supporting-information`.

Response: The section Supplementary information, with the captions of the online resources, has been included in the main document, see lines 516-538.

Concern 7: Please review your reference list to ensure that it is complete and

correct. If you have cited papers that have been retracted, please include the rationale for doing so in the manuscript text, or remove these references and replace them with relevant current references. Any changes to the reference list should be mentioned in the rebuttal letter that accompanies your revised manuscript. If you need to cite a retracted article, indicate the article's retracted status in the References list and also include a citation and full reference for the retraction notice.

Response: To address one of the reviewer's concerns, we performed statistical analyses, where we used two new libraries. To comply with the terms and conditions of these packages, we included the appropriate references. These are numbered [31] and [32], respectively.

Reviewer 1

The paper presents an approach to designing and training models for medical image registration. The authors employ multiple concepts and techniques to prepare and validate their experiments. The manuscript is well-written and generally clear in reception. I like the discussion in particular, it sounds fair and reasonable. My remarks are as follows:

Concern 1: *The paper title is a bit pretentious. The title of likely every research involving ablation study on machine learning training settings and/or hyperparameters could start with "Train smarter, not harder". I suggest focusing on the actual scope of the study, especially since the workflow covers not only abdominal CT data.*

Response: We agree with the reviewer that the original manuscript title was inappropriate, and have therefore rewritten it to better summarise our work. The revised manuscript title reads: *'Learning deep abdominal CT registration through adaptive loss weighting and synthetic data generation'*.

Concern 2: *Congratulations to the Authors on the successful implementation of the augmentation scheme, but the paper does not convince me that this is a significant contribution. The argument (repeated multiple times) about no need to store the data after augmentation on disk is not convincing too. As far as I know, the training schemes in multiple environments offer the same thing by default.*

Response: Thank you for your comment. Training deep image registration models generally requires an extensive dataset, as any deep learning model.

In our case, each training sample involves multiple (two to four) volumetric images. If these images were to be generated and stored prior to the training phase, the amount of augmentations would be limited by the available space in the computer. This is not scalable, especially as one could in theory generate infinite number of artificial copies. Having the augmentation layer is therefore extremely beneficial. Frameworks such as TensorFlow and PyTorch, as well as tailored frameworks for medical image registration such as VoxelMorph, have great functionality for creating high-performance augmentations, especially for 2D images. However, 3D medical data need to be handled with care, and it is a challenge to develop an augmentation pipeline that does not significantly impact the training runtime. Note that the augmentation layer also reads and preprocesses the data on-the-fly. We demonstrated negligible delay in training runtime and increase in GPU memory usage in adding the augmentation layer to the pipeline, as shown in Fig. S1. To the best of our knowledge, we have not seen any prior work achieving comparable results on similar 3D medical image analysis tasks. Furthermore, by implementing our augmentation scheme as a standalone TensorFlow layer, the scientific community can easily benefit from it in future work. We believe this constitutes a great contribution in itself as we have not found similar high-performance 3D data augmentation pipelines supporting non-rigid transforms. Further clarification has been made in the contributions list (lines 69-72).

Concern 3: The reader may be interested in some methodology details, e.g., how do you interpolate the data in resampling and resizing (lines 90-91)?

Response: We have added more relevant details and clarifications in lines 107-109, 208, and 211-213. Other implementation details can be found from the provided source code.

Concern 4: I'm confused with the description in lines 119-120: convolution filters are a part of convolutional layers, not max-pooling blocks.

Response: We agree with the reviewer that this was confusing. We have therefore replaced the usage of max pooling blocks with contraction blocks, where the contraction block contains a convolution, LeakyReLU activation, and a max pooling operation (cf. lines 135-137).

Concern 5: Please describe N and M in Eq. (1).
Response: We have made modifications to clarify what the variables N and M mean, see lines 155-156.

Concern 6: Please provide units for metrics like HD or TRE. Are these millimeters or pixel/voxel-size-based?

Response: The unit of these metrics were in millimetres. To address this, we have made modifications to lines 212 and 215.

Concern 7: Lines 280+: yes, I support the statement on the necessity to collect expert delineations for a reliable evaluation. I even think that with a generally scarce dataset, the Authors could put some effort into annotating a small portion of the data (5-10 cases?) and report the results in the current manuscript. Please consider such an improvement.

Response: For the Oslo-CoMet dataset, the liver parenchymas of 60 CTs had already been manually annotated for this study, and used for the statistical analysis. The vessels were automatically generated for our study, as it is an extremely challenging and tedious structure to annotate for an entire 3D volume and requires clinical expertise. This has been further clarified in the Methods section, see lines 98-100. The vessels of the 11 CTs from the Oslo-CoMet test set have now been manually delineated, which leads to an added comparison study between the manual and automatic generations. However, a large inter-rater variability has been observed from this additional study, between the manual annotator and the automatic segmentation model. To better explore the impact of annotation differences on the final performance, manual delineations would be required as well for the training set. While we agree with the reviewer on the relevance of such investigation, the manual and time-consuming work required for annotating the entire dataset, coupled to model retraining, prevent inclusion in this study. Therefore such analysis has been mentioned as an interesting prospect to explore in future work. See lines 356-361, and 363-364.

Concern 8: Change formatting of axis tick labels in Figs. S6-S13. The loss weights look weird in a "4.50e-1" format, it's just "0.45".

Response: We agree with the reviewer, and have changed the tick labels to decimal notation for all the figures the reviewer suggested.

Reviewer 2

This paper investigates different methods to improve the registration of medical images using convolutional neural networks. Different training strategies, loss functions, and transfer learning schemes are examined. The paper focuses on the registration of sparse medical data using deep learning. It is an important step

in the development of new machine-learning-based methods in the medical field.

The framework used (based on VoxelMorph), the U-Net and the use of the segmentations during training are explained very clearly and precisely, so that it is also easy to implement. Figure 1 is also very well-structured and thus helps a lot in understanding the framework used.

Some additional comments are listed as follows:

Concern 1: You work on deformable image registration. However, the word "deformable" did not appear anywhere in your paper. My advice would be to mention the term "deformable" in the beginning of the paper, so that it is clear which transformation (rigid, affine, deformable,...) you are dealing with - for example see your source [9].

Response: We thank the reviewer for the comment. We have specified the type of deformation our works deals with in both the abstract and at the start of the introduction. See the point Purpose in the abstract and lines 13-19, 50-51, and 65.

Concern 2: Section Results: In tables 2-5, one more column could be added: Values of the metrics before registration - to better examine the potential of deep learning image registration and to check if all models succeeded in improving the values of the unregistered data (image-based and segmentation-based similarity metrics).

Response: We agree with the reviewer that this is a good idea, and have computed the metrics for the unregistered data and added these to Tables 2 to 5 in a new row named "Unregistered".

Concern 3: I would also recommend to do a significance test and to mark the results in tables 2-5.

Response: We agree with the reviewer that adding hypothesis tests would strengthen the paper. We have therefore performed five sets of statistical tests to assess: 1) performance contrasts between designs, 2) benefit of transfer learning, 3) benefit of segmentation-guiding, 4) benefit of uncertainty weighting, and 5) performance contrasts between the baseline and segmentation-guided models and the traditional methods in ANTs. In 1), we performed multiple comparisons of all contrasts. Due to the small sample size of the Oslo-CoMet dataset, we only performed this on the IXI dataset. The rest of the tests, 2)-5), were conducted on the Oslo-CoMet dataset only, as that was our main use case. Four paragraphs

describing the statistical analysis process were included to the text (cf. lines 218-243). In addition, additional results from the statistical analysis were added to the Results section (see lines 253-261 and 268-277). However, the results were not marked in the tables themselves, but rather solely described in the Results section and Supplementary Materials. The discussion of these results has been also included in the Discussion section, see lines 303-305, 209-314, 335-344, and 356-364.
